# The Prognostic Value of EDRIC and Its Association With Radiation‐Induced Lymphopenia in Patients With Esophageal Cancer Undergoing Radical Radiotherapy: A Single‐Center Chinese Study

**DOI:** 10.1002/kjm2.70251

**Published:** 2026-06-11

**Authors:** Yue Ke, Chen Yang, Yu‐Xing Li, Xiao‐Xiao Liu, Xiao Liu, Jia‐Min Gao, Hong‐Bing Ma

**Affiliations:** ^1^ Department of Radiation Oncology The Second Affiliated Hospital of Xi'an Jiaotong University Xi'an China

**Keywords:** definitive radiotherapy, EDRIC, esophageal cancer, lymphopenia, prognosis

## Abstract

This study aimed to evaluate the predictive value of the estimated dose of radiation to immune cells (EDRIC) in patients with esophageal cancer receiving radical radiotherapy. We retrospectively reviewed the data of patients with esophageal cancer who underwent definitive radiotherapy at our institution. The Kaplan–Meier method and log‐rank test were used to assess the survival curves. Univariate and multivariate Cox regression analyses were performed to identify the prognostic factors associated with overall survival (OS) and progression‐free survival (PFS). Additionally, binary logistic regression analyses were used to identify factors associated with severe lymphopenia. A total of 166 patients, with a median follow‐up duration of 59 months, were evaluated. The median EDRIC was 7.23 Gy. The 1‐, 3‐, and 5‐year OS rates were 81.9%, 49.4%, and 33.7% in the EDRIC < 7.23 Gy group, compared with 62.7%, 21.7%, and 19.3% in the EDRIC ≥ 7.23 Gy group (*p* < 0.001). Prognostic analysis identified Eastern Cooperative Oncology Group performance status, TNM stage, treatment, EDRIC, and lymphopenia as significant independent prognostic factors of OS (*p* < 0.05). Higher EDRIC, N stage, and lymphopenia were identified as independent risk factors for PFS. Logistic regression analysis indicated that treatment and EDRIC were significantly associated with severe lymphopenia (*p* < 0.05). EDRIC serves not only as a prognostic marker for radical radiotherapy in esophageal cancer, but also as a potential biomarker for radiation‐induced lymphopenia.

**Abbreviations**
3D‐CRT: three‐dimensional conformal radiation therapy
ALC: absolute lymphocyte count
CBC: complete blood counts
CCRT: concurrent chemoradiotherapy
CI: confidence interval
CRT: chemoradiotherapy
CTV: clinical target volume
DVH: dose‐volume histogram
ECOG: Eastern Cooperative Oncology Group
EDRIC: estimated dose of radiation to immune cells
ENI: elective node irradiation
GTV: gross target volume
HR: hazard ratio
IFI: involved field irradiation
IMRT: intensity modulated radiation therapy
MHD: mean heart dose
MLD: mean lung dose
mOS: median overall survival
mPFS: median progression‐free survival
N: lymph node
NSCLC: non‐small cell lung cancer
OR: odds ratio
OS: overall survival
SCC: squamous cell carcinoma
SCRT: sequential chemoradiotherapy

## 1 Introduction

Esophageal cancer is one of the most common and aggressive malignant tumors, ranking as the seventh highest in incidence and sixth highest in mortality worldwide [1]. China has a high incidence of esophageal cancer, with an estimated 477,900 new cases and 375,000 deaths in 2015 [2]. More than 90% of the cases in China are squamous cell carcinoma, followed by adenocarcinoma [3]. In the United States, the 5‐year relative survival rate across all stages of esophageal cancer was 20% in 2022 [4]. Radio‐ and chemotherapy remain the mainstay treatments for unresectable esophageal cancers. Over the past few decades, immunotherapy has emerged as a novel treatment strategy that has been widely adopted for various cancer types, including esophageal cancer. A combination of immune checkpoint inhibitors (ICIs) and chemotherapy has become the first‐line treatment for metastatic esophageal cancer [5, 6]. Furthermore, chemoradiotherapy combined with ICIs may extend the progression‐free survival (PFS) and overall survival (OS) in patients with programmed death‐ligand 1 positive, unresectable advanced esophageal squamous cell carcinoma [7]. However, the effects of radiotherapy on the immune system are complex.

Radiotherapy (RT) can promote tumor‐specific antigen release, activate antigen‐presenting dendritic cells and T cells, and initiate acquired anti‐tumor immune responses. When combined with radio‐ and immunotherapy, this acquired anti‐tumor immune response is continuously activated, resulting in a synergistic effect [8, 9, 10, 11]. In addition, immunotherapy combined with radiotherapy can enhance systemic immune responses, a phenomenon known as the abscopal effect [12, 13]. However, radiation can also exert deleterious effects on the immune system by depleting immune cells, especially cytotoxic T lymphocytes, which may reduce the efficacy of both radio‐ and immunotherapy [14, 15, 16, 17].

Therefore, quantifying the impact of radiation on the immune system has become an area of increasing interest. In a secondary analysis of the RTOG0617 study, Jin et al. hypothesized that the immune system is “an organ at risk” and found that the effective radiation dose to the immune cells (EDIC) was associated with prognosis in patients with non‐small cell lung cancer (NSCLC) [18]. Further optimization of the estimated dose of radiation to immune cells (EDRIC), using the number of radiation fractions, mean heart dose (MHD), mean lung dose (MLD), and mean body dose (MBD), showed a significant correlation with prognosis in stage III NSCLC [19]. Although recent studies have investigated the prognostic value of EDRIC in patients with esophageal cancer, the findings remain inconsistent [20, 21]. Therefore, the prognostic value of EDRIC in esophageal cancer warrants further investigation. Additionally, its potential effect on lymphocyte counts remains unclear. Peripheral lymphopenia, a common side effect of radiation, has been associated with poor prognosis in solid malignancies [22]. However, whether EDRIC influences peripheral blood lymphocyte counts and, in turn, affects patient prognosis needs to be determined.

Therefore, this study aimed to investigate the prognostic value of EDRIC in patients with esophageal cancer undergoing radical radiotherapy, clarify the relationship between EDRIC and lymphocytopenia, and assess EDRIC as a potential biomarker for radiation‐induced immune system damage in these patients.

## 2 Patients and Methods

### 2.1 Patients Selection

The records of patients with esophageal cancer who received radiotherapy at our institution between January 2013 and December 2022 were retrospectively reviewed. The inclusion criteria were as follows: (1) age > 18 years; (2) histologically confirmed esophageal cancer according to the American Joint Committee on Cancer, eighth edition; (3) receipt of conventional fractionated radical radiotherapy (≥ 50 Gy) without unplanned interruption, defined as any unscheduled interruption of radiotherapy exceeding seven consecutive days; and (4) retrievable dosimetric records and complete blood counts. The exclusion criteria were as follows: (1) history of surgery or radiotherapy; (2) prior immunotherapy or chemotherapy; (3) history of tumor or autoimmune disease; (4) presence of distant metastases; and (5) Eastern Cooperative Oncology Group (ECOG) performance status > 2.

### 2.2 Radiation Treatment

All patients underwent a CT scan of 0.5 cm slice thickness, extending from the bilateral neck and supraclavicular regions to the thorax and upper abdomen, in the supine position. The gross target volume (GTV) included the primary tumor (GTVp) and lymph node metastasis (GTVn). The clinical target volume (CTV) of the primary tumor (CTVp) was defined as a 3.0 cm superior and inferior margin and a 0.6 cm lateral margin from the GTVp. The CTV for lymph node metastasis (CTVn) was defined as the region containing the GTVn. The planning target volume was generated by adding a 0.5 cm margin in each direction of the CTV. A total dose of 50–70 Gy was prescribed and delivered in 25–35 fractions, at a frequency of five fractions per week.

### 2.3 Chemotherapy Regimen

Chemotherapy regimens included TP, PF, and single‐agent therapy. TP regimen consisted of paclitaxel 135–175 mg/m^2^ + cisplatin 75 mg/m^2^ on Day 1, administered every 3 weeks. The PF regimen comprised cisplatin 75 mg/m^2^ on Day 1 + fluorouracil 800 mg/m^2^ on Day 1–4, administered every 4 weeks. Single‐agent therapy includes capecitabine (625 mg/m^2^ twice daily on Days 1–5, 8–12, 15–19) every 3 weeks, or S‐1 (40 mg/m^2^ twice daily on Days 1–14) administered every 3 weeks.

### 2.4 Data Collection

Data on clinicopathological variables, peripheral complete blood count (CBC), serum albumin levels, and radiation dosimetry were collected from the medical records. The clinicopathological variables included age, sex, ECOG performance status, chemotherapy regimen, smoking history, drinking history, TNM stage, pathology, primary tumor location, and degree of differentiation. The treatment approaches included concurrent chemoradiotherapy (CCRT), sequential chemoradiotherapy (SCRT), and radiotherapy.

CBC was performed every week during radiotherapy. Radiation parameters including radiation technique, number of radiation fractions, MHD, MLD, and MBD, were collected based on dose‐volume histograms (DVH) from the treatment planning system (Varian Eclipse 13.6). The MBD was calculated over the region from the first cervical vertebra to the fifth lumbar vertebra. The EDRIC model was developed by Jin et al. and further refined by Ladbury et al. [18, 19] In this study, we calculated the EDRIC using MLD, MHD, MBD, and the number of radiation fractions.

The model was as follows:

$$\begin{array}{cc} \text{EDRIC}= & \;0.12\times \mathrm{MLD}+0.08\times \mathrm{MHD}\\ & \;+\left[0.45+0.35\times 0.85\times {\left(\frac{\#\text{of fractions}}{45}\right)}^{\frac{1}{2}}\right]\\ & \;\times \mathrm{MBD} \end{array}$$

### 2.5 Definition of Lymphopenia

According to the Common Terminology Criteria for Adverse Events version 5.0, lymphopenia was defined as an absolute lymphocyte count (ALC) < 1.00 × 10^9^/L. The grading was as follows: Grade 1: < 0.8 × 10^9^/L, Grade 2: < 0.8–0.5 × 10^9^/L, Grade 3: < 0.5–0.2 × 10^9^/L, Grade 4: < 0.2 × 10^9^/L. Severe lymphopenia was defined as Grade 3–4 lymphopenia. In this study, lymphopenia refers to the nadir (lowest recorded lymphocyte count) during the course of radical radiotherapy.

### 2.6 Statistical Analyzes

The median follow‐up time was estimated using the reverse Kaplan–Meier method. OS was defined as the time from radiotherapy initiation to death from any cause, whereas PFS was defined as the time from radiotherapy initiation to the first documented disease progression or death from any cause. The Kaplan–Meier method was used to estimate survival, and the log‐rank test was used to compare survival curves. EDRIC was dichotomized and analyzed using the Kaplan–Meier method. Univariate and multivariate Cox proportional hazards regression analyses were used to evaluate the associations between potential prognostic factors, OS, and PFS. Covariates with *p* values < 0.1 in the univariate analysis were entered into the multivariate analysis. A stepwise forward selection approach was used for the final multivariate analysis. Continuous variables were dichotomized based on their median values. Hazard ratios (HRs) were derived from the regression coefficients. Additionally, univariate and multivariate binary logistic regression analyses were conducted to identify factors associated with severe lymphopenia, with odds ratios (ORs) estimated using regression analysis. Statistical analyses were performed using SPSS version 26.0 and GraphPad Prism version 9.3.0 software (GraphPad Software Inc.).

## 3 Results

### 3.1 Patient Characteristics

A total of 166 patients were enrolled in this study, including 122 (73.5%) men and 44 (26.5%) women. The median patients age was 70 years (range, 41–94) years. A history of smoking and alcohol consumption was reported in 72 (43.4%) and 50 (30.1%) patients, respectively. Most patients (98.2%) had squamous cell carcinoma on histological examination, with 6 (3.6%) patients at Stage I, 52 (31.3%) at Stage II, 61 (36.7%) at Stage III, and 47 (28.3%) at Stage IVA. Of these, 84 (50.6%) patients received CCRT, 53 (31.9%) underwent SCRT, and 29 (17.5%) received radiotherapy alone. All patients received radiotherapy using either three‐dimensional conformal radiation therapy (3D–CRT; 22.9%) or intensity‐modulated radiation therapy (IMRT; 77.1%) at a median dose of 60 Gy (range 50–70). The median EDRIC was 7.23 Gy. The rates of lymphopenia were 6.6%, 14.5%, 61.4%, and 17.5% for Grades 1, 2, 3, and 4, respectively. The clinicopathological characteristics of the patients are listed in Table 1.

**TABLE 1:** Clinicopathological characteristics of the patients.

Characteristic	No. of patients
	*N* = 166
Gender	
Male	122 (73.5%)
Female	44 (26.5%)
Age, years	
< 70	82 (49.4%)
≧ 70	84 (50.6%)
Smoking history	
No	94 (56.6%)
Yes	72 (43.4%)
Drinking history	
No	116 (69.9%)
Yes	50 (30.1%)
ECOG	
0–1	86 (51.8%)
2	80 (48.2%)
Pathology	
Not SCC	3 (1.8%)
SCC	163 (98.2%)
Tumor location	
Cervical	7 (4.2%)
Upper	43 (25.9%)
Middle	54 (32.5%)
Lower	62 (37.3%)
Differentiation	
High	43 (25.9%)
Moderate	96 (57.8)
Low	27 (16.3%)
T	
1	7 (4.2%)
2	51 (30.7%)
3	65 (39.2%)
4	43 (25.9%)
N	
0	33 (19.9%)
1	65 (39.2%)
2	60 (36.1%)
3	8 (4.8%)
TNM	
I	6 (3.6%)
II	52 (31.3%)
III	61 (36.7%)
IVA	47 (28.3%)
Treatment	
CCRT	84 (50.6%)
SCRT	53 (31.9%)
RT	29 (17.5%)
Chemotherapy regimen	
TP	71 (42.8%)
PF	31 (18.7%)
S1/Capecitabine	35 (21.1%)
No	29 (17.5%)
Radiotherapy technology	
IMRT	128 (77.1%)
3D‐CRT	38 (22.9%)
Radiation dose	
< 60 Gy	87 (52.4%)
≥ 60 Gy	79 (47.6%)
EDRIC, Gy	
≤ 7.23	83 (50%)
> 7.23	83 (50%)
Lymphopenia	
G1	11 (6.6%)
G2	24 (14.5%)
G3	102 (61.4)
G4	29 (17.5)
Hypoalbuminemia	
No	110 (66.3%)
Yes	56 (33.7%)

Abbreviations: 3D‐CRT, three‐dimensional conformal radiation therapy; CCRT, concurrent chemoradiotherapy; ECOG, Eastern Cooperative Oncology Group; EDRIC, estimated dose of radiation to immune cells; IMRT, intensity modulated radiation therapy; PF, cisplatin + fluorouracil; RT, radiotherapy; SCC, squamous cell carcinoma; SCRT, sequential chemoradiotherapy; TP, paclitaxel + cisplatin.

### 3.2 Overall Survival and Progression‐Free Survival

**FIGURE 1:**
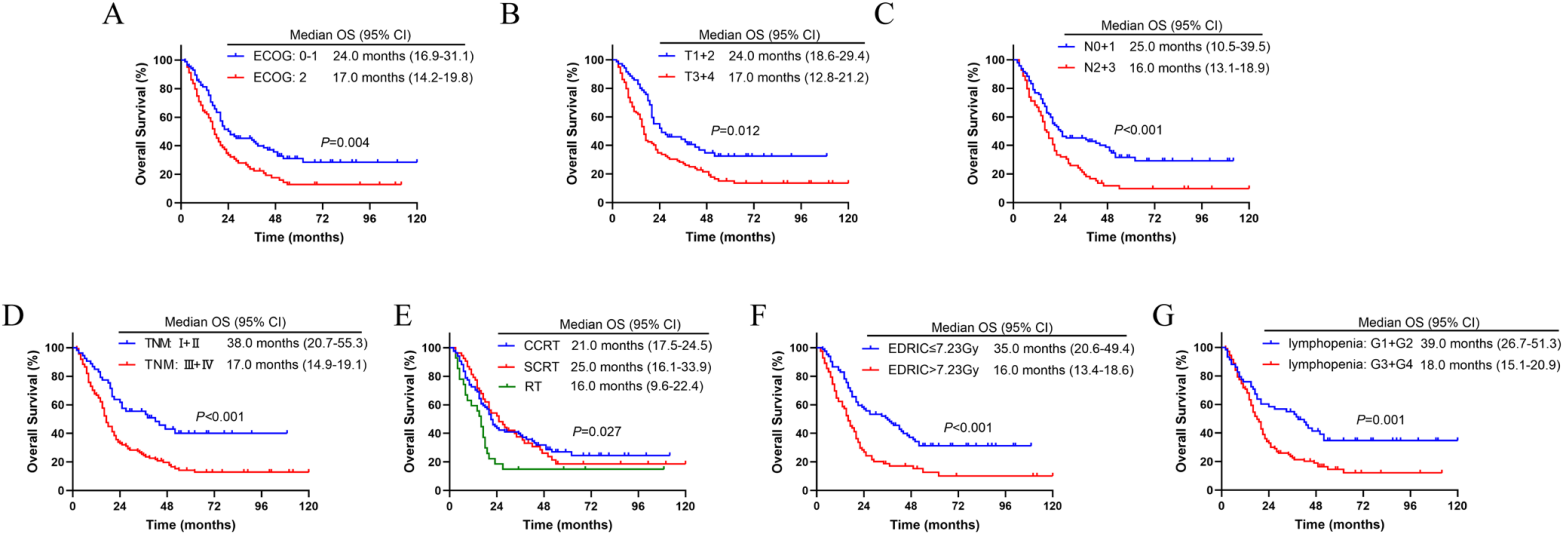
Kaplan–Meier curves of overall survival (OS) for the entire cohort stratified by (A) ECOG, (B) T stage, (C) N stage, (D) TNM stage, (E) treatment, (F) EDRIC, and (G) lymphopenia. ECOG, Eastern Cooperative Oncology Group; EDRIC, estimated dose of radiation to immune cells.

The median follow‐up time for the entire cohort was 59 months (range, 1–120 months), with all patients followed up until death or the last available follow‐up. The median OS (mOS) was 20 months. The 1‐, 3‐, and 5‐year OS rates were 81.9%, 49.4%, and 33.7% in the EDRIC < 7.23 Gy group, compared with 62.7%, 21.7%, and 19.3% in the EDRIC ≥ 7.23 Gy group, respectively. Stratified analysis by EDRIC showed an mOS of 35.0 months (95% confidence interval [CI], 20.6–49.4 months) for the EDRIC < 7.23 Gy group and 16.0 months (95% CI, 13.4–18.6 months) for the EDRIC ≥ 7.23 Gy group, with a statistically significant difference between the two subgroups (log‐rank *p* < 0.001). Additionally, ECOG performance status, T stage, N stage, TNM stage, treatment, EDRIC, and lymphopenia were significantly associated with OS (all *p* < 0.05, Figure 1). The mOS and corresponding 95% CI for each subgroup stratified by these factors are presented in Figure 1.

**FIGURE 2:**
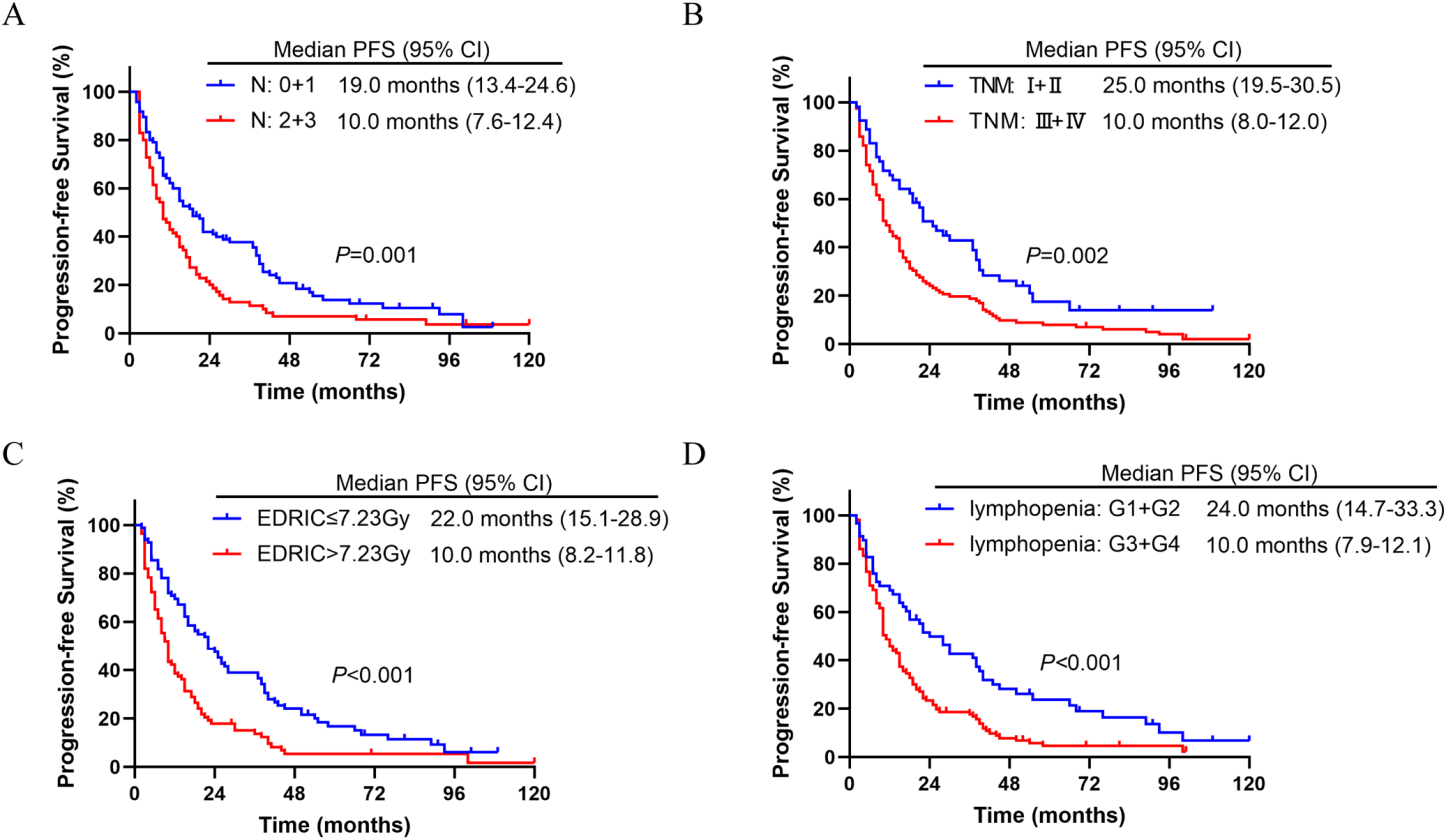
Kaplan–Meier curves of progression‐free survival (PFS) for the entire cohort, stratified by (A) N stage, (B) TNM stage, (C) EDRIC, and (D) lymphopenia. EDRIC, estimated dose of radiation to immune cells.

The median PFS (mPFS) for the entire cohort was 15 months. When stratified by EDRIC, the mPFS was 22.0 months (95% CI, 15.1–28.9 months) in the EDRIC < 7.23 Gy group and 10.0 months (95% CI, 8.2–11.8 months) in the EDRIC ≥ 7.23 Gy group. Kaplan–Meier analysis demonstrated that N stage, TNM stage, EDRIC, and lymphopenia were significantly associated with PFS (all *p* < 0.05; Figure 2). The mPFS with 95% CIs, and corresponding *p*‐values are presented in Figure 2.

### 3.3 Univariate and Multivariate Analyses

Univariate analysis showed that ECOG performance status (*p* = 0.005), T stage (*p* = 0.014), N stage (*p* < 0.001), TNM stage (*p* < 0.001), treatment (CCRT vs. radiotherapy, *p* = 0.012; SCRT vs. radiotherapy, *p* = 0.026), EDRIC (*p* < 0.001), and lymphopenia (*p* = 0.001) were significantly associated with OS. In multivariate analysis, ECOG performance status (HR = 0.649, 95% CI: 0.439–0.960; *p* = 0.03), TNM stage (HR = 0.614, 95% CI: 0.405–0.929; *p* = 0.021), treatment (CCRT vs. radiotherapy, HR = 0.562, 95% CI: 0.336–0.941; *p* = 0.028; SCRT vs. radiotherapy, HR = 0.532, 95% CI: 0.318–0.889; *p* = 0.016), EDRIC (HR = 0.660, 95% CI: 0.446–0.976; *p* = 0.037), and lymphopenia (HR = 0.596, 95% CI: 0.391–0.906; *p* = 0.015) were identified as independent prognostic factors of OS in esophageal cancer (Table 2).

**TABLE 2:** Univariate and multivariate analyses of clinical factors associated with overall survival.

Variables	Univariate			Multivariate		
	HR	95% CI	*p*	HR	95% CI	*p*
Gender						
Female versus male	1.045	0.699–1.564	0.829			
Age, years						
< 70 versus ≥ 70	0.731	0.513–1.042	0.083			0.189
Smoking						
No versus yes	1.274	0.891–1.822	0.184			
Drinking						
No versus yes	1.010	0.691–1.476	0.959			
Histology						
Not SCC versus SCC	1.408	0.447–4.438	0.559			
Tumor location						
Cervical + upper versus middle + lower	0.757	0.508–1.128	0.172			
Differentiation						
High versus low	0.686	0.397–1.186	0.177			
Moderate versus low	0.923	0.581–1.466	0.734			
ECOG						
0–1 versus 2	0.603	0.423–0.860	0.005	0.649	0.439–0.960	0.030
T						
1 + 2 versus 3 + 4	0.614	0.416–0.905	0.014			0.484
N						
0 + 1 versus 2 + 3	0.523	0.366–0.746	0.000			0.231
TNM						
I + II versus III + IVA	0.481	0.324–0.716	0.000	0.614	0.405–0.929	0.021
Treatment						
CCRT versus RT	0.543	0.337–0.875	0.012	0.562	0.336–0.941	0.028
SCRT versus RT	0.562	0.338–0.934	0.026	0.532	0.318–0.889	0.016
Radiotherapy technology						
IMRT versus 3D‐CRT	0.959	0.639–1.441	0.842			
Radiation dose						
< 60 versus ≥ 60 Gy	1.237	0.869–1.761	0.239			
EDRIC, Gy						
≤ 7.23 versus > 7.23	0.500	0.349–0.717	0.000	0.660	0.446–0.976	0.037
Lymphopenia						
G1 + G2 versus G3 + G4	0.524	0.354–0.777	0.001	0.596	0.391–0.906	0.015
Hypoalbuminemia						
No versus yes	1.403	0.955–2.062	0.085			0.054

Abbreviations: 3D‐CRT, three‐dimensional conformal radiation therapy; CCRT, concurrent chemoradiotherapy; CI, confidence interval; ECOG, Eastern Cooperative Oncology Group; EDRIC, estimated dose of radiation to immune cells; HR, hazards ratio; IMRT, intensity modulated radiation therapy; RT, radiotherapy; SCC, squamous cell carcinoma; SCRT, sequential chemoradiotherapy.

Univariate analysis showed that N stage (*p* = 0.002), TNM stage (*p* = 0.002), EDRIC (*p* < 0.001), and lymphopenia (*p* = 0.001) were prognostic factors of PFS. Multivariate analysis showed that N stage (HR = 0.690, 95% CI: 0.493–0.965; *p* = 0.03), EDRIC (HR = 0.670, 95% CI: 0.474–0.946; *p* = 0.023), and lymphopenia (HR = 0.629, 95% CI: 0.438–0.903; *p* = 0.012) were independent risk factors for PFS (Table 3).

**TABLE 3:** Univariate and multivariate analyses of clinical factors associated with progression‐free survival.

Variables	Univariate			Multivariate		
	HR	95% CI	*p*	HR	95% CI	*p*
Gender						
Female versus male	1.044	0.725–1.503	0.817			
Age, years						
< 70 versus ≥ 70	0.844	0.614–1.162	0.300			
Smoking						
No versus yes	1.168	0.847–1.611	0.344			
Drinking						
No versus yes	1.037	0.731–1.469	0.840			
Histology						
Not SCC versus SCC	1.085	0.345–3.416	0.889			
Tumor location						
Cervical + upper versus middle + lower	0.918	0.648–1.300	0.630			
Differentiation						
High versus low	0.742	0.451–1.221	0.240			
Moderate versus low	0.815	0.524–1.268	0.364			
ECOG						
0–1 versus 2	0.769	0.559–1.060	0.109			
T						
1 + 2 versus 3 + 4	0.722	0.512–1.018	0.063			0.232
N						
0 + 1 versus 2 + 3	0.598	0.432–0.828	0.002	0.690	0.493–0.965	0.030
TNM						
I + II versus III + IVA	0.584	0.412–0.826	0.002			0.298
Treatment						
CCRT versus RT	0.691	0.444–1.075	0.101			
SCRT versus RT	0.699	0.436–1.120	0.137			
Radiotherapy technology						
IMRT versus 3D‐CRT	1.106	0.751–1.628	0.612			
Radiation dose						
< 60 versus ≥ 60 Gy	1.191	0.866–1.639	0.282			
EDRIC, Gy						
≤ 7.23 versus > 7.23	0.542	0.392–0.750	0.000	0.670	0.474–0.946	0.023
Lymphopenia						
G1 + G2 versus G3 + G4	0.543	0.384–0.769	0.001	0.629	0.438–0.903	0.012
Hypoalbuminemia						
No versus yes	1.208	0.861–1.695	0.275			

Abbreviations: 3D‐CRT, three‐dimensional conformal radiation therapy; CCRT, concurrent chemoradiotherapy; CI, confidence interval; ECOG, Eastern Cooperative Oncology Group; EDRIC, estimated dose of radiation to immune cells; HR, hazards ratio; IMRT, intensity modulated radiation therapy; RT, radiotherapy; SCC, squamous cell carcinoma; SCRT, sequential chemoradiotherapy.

### 3.4 Subgroup Analyses of the Prognostic Value of EDRIC

**FIGURE 3:**
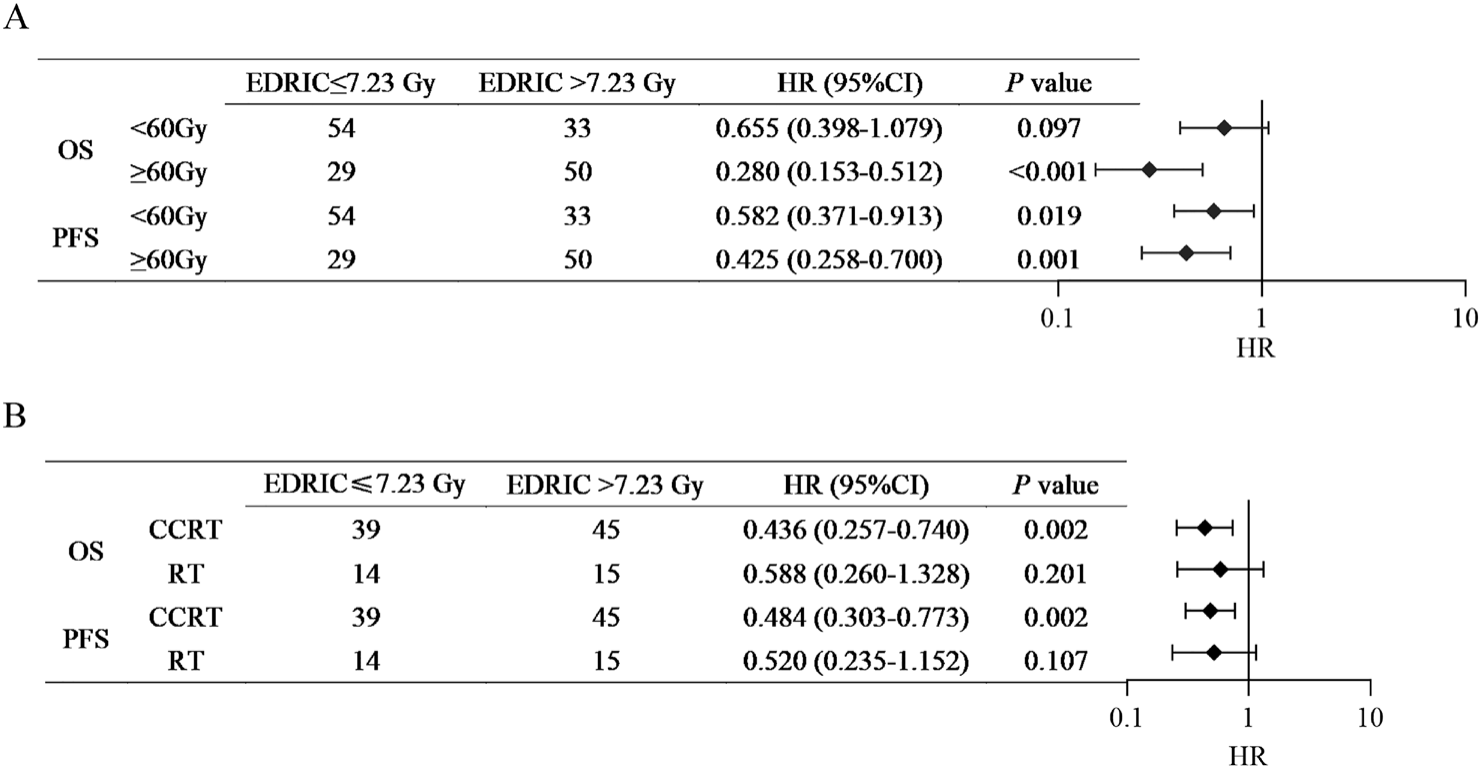
Forest plots of subgroup analyses for OS and PFS. HRs and 95% CIs for EDRIC (≤ 7.23 vs. > 7.23 Gy) are shown for each subgroup defined by (A) radiation dose (< 60 vs. ≥ 60 Gy) and (B) treatment modality (CCRT vs. RT alone). CCRT, concurrent chemoradiotherapy; CI, confidence interval; EDRIC, estimated dose of radiation to immune cells; HR, hazard ratio; OS, overall survival; PFS, progression‐free survival; RT, radiotherapy.

Subgroup analyses were performed stratified by radiation dose and treatment modality. In the < 60 Gy subgroup (*n* = 87), low EDRIC (≤ 7.23 Gy) was associated with a trend toward improved OS (HR = 0.655, 95% CI: 0.398–1.079; *p* = 0.097) and significantly improved PFS (HR = 0.582, 95% CI: 0.371–0.913; *p* = 0.019). In the ≥ 60 Gy subgroup (*n* = 79), low EDRIC was associated with significantly improved OS (HR = 0.280, 95% CI: 0.153–0.512; *p* < 0.001) and PFS (HR = 0.425, 95% CI: 0.258–0.700; *p* = 0.001) (Figure 3A). In the CCRT subgroup (*n* = 84), a low EDRIC was associated with significantly improved OS (HR = 0.436, 95% CI: 0.257–0.740; *p* = 0.002) and PFS (HR = 0.484, 95% CI: 0.303–0.773; *p* = 0.002). In the radiotherapy‐alone subgroup (*n* = 29), low EDRIC showed directionally consistent but nonsignificant associations with OS (HR = 0.588, 95% CI: 0.260–1.328; *p* = 0.201) and PFS (HR = 0.520, 95% CI: 0.235–1.152; *p* = 0.107), which may be attributed to the limited sample size (Figure 3B).

### 3.5 Interaction Between EDRIC and Treatment Modality

To assess whether the prognostic effect of EDRIC varied by treatment type, an interaction term (EDRIC × treatment) was included in the multivariate Cox regression models for OS and PFS. The interaction was not statistically significant for either OS (*p* = 0.971) or PFS (*p* = 0.777), indicating that the association between EDRIC and survival did not vary significantly between patients who received CCRT and those who received radiotherapy alone (Table S1). Therefore, the previously observed lack of significance in the radiotherapy‐alone subgroup may be attributed to the limited sample size (*n* = 29) rather than the absence of an effect.

In conclusion, the prognostic value of EDRIC for OS and PFS in patients with esophageal cancer may be influenced by radiotherapy dose and treatment modality, with more pronounced associations observed in patients receiving higher radiation doses (≥ 60 Gy) or CCRT.

### 3.6 Logistic Regression Analysis of Severe Lymphopenia

Among the 166 patients, 131 (78.9%) experienced severe lymphopenia (Grade 3 and 4) during radiotherapy. Univariate and multivariate logistic regression analyses were performed to identify factors associated with severe lymphopenia. The results showed that the treatment type (CCRT vs. radiotherapy: OR = 2.599, 95% CI: 1.070–6.317; *p* = 0.035) and EDRIC (≤ 7.23 Gy vs. > 7.23 Gy: OR = 0.409, 95% CI: 0.209–0.800; *p* = 0.009) were significantly associated with severe lymphopenia during radiotherapy (Table 4).

**TABLE 4:** Logistic regression analysis of severe lymphopenia.

Variables	Univariate			Multivariate		
	OR	95% CI	*p*	OR	95% CI	*p*
Gender						
Female versus male	0.827	0.397–1.724	0.613			
Age, years						
< 70 versus ≥ 70	1.643	0.862–3.132	0.131			
Smoking						
No versus yes	1.684	0.884–3.206	0.113			
Drinking						
No versus yes	1.069	0.535–2.137	0.851			
Tumor location						
Cervical + upper versus middle + lower	1.061	0.528–2.133	0.868			
Differentiation						
High versus low	1.283	0.476–3.458	0.622			
Moderate versus low	1.375	0.572–3.306	0.477			
ECOG						
0–1 versus 2	1.713	0.900–3.262	0.101			
T						
1 + 2 versus 3 + 4	0.818	0.421–1.590	0.554			
N						
0 + 1 versus 2 + 3	0.587	0.301–1.142	0.117			
TNM						
I + II versus III + IVA	0.651	0.336–1.262	0.203			
Treatment						
CCRT versus RT	2.529	1.064–6.009	0.036	2.599	1.070–6.317	0.035
SCRT versus RT	2.083	0.827–5.248	0.119	2.330	0.899–6.039	0.082
Radiotherapy technology						
IMRT versus 3D‐CRT	0.823	0.380–1.783	0.621			
Radiation dose						
< 60 versus ≥ 60 Gy	0.843	0.444–1.600	0.602			
EDRIC, Gy						
≤ 7.23 versus > 7.23	0.421	0.218–0.813	0.010	0.409	0.209–0.800	0.009
Hypoalbuminemia						
No versus yes	0.829	0.419–1.640	0.590			

Abbreviations: 3D‐CRT, three‐dimensional conformal radiation therapy; CCRT, concurrent chemoradiotherapy; CI, confidence interval; ECOG, Eastern Cooperative Oncology Group; EDRIC, estimated dose of radiation to immune cells; OR, odds ratio; IMRT, intensity modulated radiation therapy; RT, radiotherapy; SCRT, sequential chemoradiotherapy.

## 4 Discussion

In this study, we found that ECOG performance status, T stage, N stage, TNM stage, treatment, EDRIC score, and lymphopenia were independent prognostic factors for OS in patients with EC undergoing definitive radiotherapy. Furthermore, the EDRIC score, N stage, TNM stage, and lymphopenia were prognostic factors for PFS. Higher EDRIC and CCRT scores were significantly associated with severe lymphopenia during radiotherapy.

During thoracic radiotherapy, the heart and lungs are recognized as primary organs at risk. Increasing radiation doses to these organs have been associated with unfavorable outcomes in NSCLC [23, 24]. The RTOG 0617 trial confirmed this observation, demonstrating that increasing the dose from the standard dose of 60 to 74 Gy did not improve survival outcomes [25]. Secondary analysis of this trial revealed that MHD and MLD were significantly associated with OS in univariate Cox regression analysis. Notably, the incidence of grade ≥ 3 pulmonary toxicity remained below 5%, with no documented cardiac mortality. Collectively, these findings imply that the association between cardiopulmonary radiation dose and survival outcomes may involve mechanisms beyond direct pulmonary and cardiac toxicity [18].

Radiation‐induced immunosuppression has been proposed as a plausible explanation for the reduced OS in the high‐dose groups, shifting research attention toward radiation‐triggered immune modulation. Supporting this concept, Jin et al.'s secondary analysis of RTOG 0617 established that EDIC correlated with both reduced OS and increased radiotherapy‐induced immune toxicity in patients with Stage III NSCLC [18]. Similarly, Ladbury et al. developed a model incorporating MBD rather than the integrated total dose divided by 62 × 10^3^, demonstrating a significant association between EDRIC and survival outcomes in patients with Stage III NSCLC [19].

Although prior studies on esophageal cancer have reported conflicting findings regarding the prognostic value of EDRIC, our results support its role as a reliable biomarker [20, 21]. Given the challenges in accurate assessment of immune system radiation exposure, we propose that circulating immune cells may be considered additional organs at risk during external beam radiotherapy. Implementing dedicated dose constraints for these cells may optimize treatment planning, minimize radiation‐induced immune damage, and provide patients with sustained immunological benefits.

Our study established that severe lymphopenia is an independent prognostic factor for both OS and PFS in the patient cohort. Although the radiotherapy‐induced reduction in ALC is a well‐documented phenomenon primarily attributed to the inherent radiosensitivity of lymphocytes [26], the clinical impact of treatment‐related lymphopenia on cancer survival outcomes remains controversial. However, substantial evidence supports a correlation between low ALC and unfavorable prognosis across various solid malignancies [27, 28]. The interpretation of lymphocyte dynamics requires the consideration of several biological factors. Lymphocytes consist of heterogeneous subpopulations with distinct radiation sensitivity profiles and possess regenerative capacity through replenishment from splenic and bone marrow reservoirs. Nutritional status significantly influences peripheral lymphocyte levels. Consequently, isolated peripheral lymphocyte counts provide an incomplete assessment of radiation‐induced immune damage [21]. Notably, our investigation revealed a significant association between elevated EDRIC values and lymphopenia, suggesting that EDRIC is a promising biomarker of radiation‐induced immune system damage during thoracic radiotherapy. Therefore, EDRIC may serve as a key indicator of radiation‐induced immune system damage in thoracic radiotherapy, given its dual effect on lymphocyte depletion during treatment and its predictive capacity for long‐term immune impairment and recovery. Additionally, we confirmed that CCRT is a significant risk factor for severe lymphopenia, consistent with the findings of Zhou et al., who identified treatment‐related lymphopenia during CCRT as an independent predictor of poor therapeutic response in esophageal squamous cell carcinoma [29]. The prognostic value of EDRIC may be confounded by the lymphotoxic effects of CCRT. However, several findings do not support this interpretation. First, EDRIC remained an independent predictor even after adjusting for treatment modality in multivariate analysis. Second, formal interaction analysis showed no significant effect modification by treatment for either OS or PFS. Third, hazard ratios in the radiotherapy‐alone subgroup were directionally consistent, although not statistically significant due to the small sample size. Collectively, these findings support the use of EDRIC as a treatment‐independent biomarker of radiation‐induced immune damage.

Furthermore, our analysis revealed no significant differences in survival based on radiation dose stratification (< 60 vs. ≥ 60 Gy) or radiotherapy techniques (3D‐CRT vs. IMRT) in patients with esophageal cancer. CCRT is the standard of care for inoperable, locally advanced disease. The RTOG 9405 trial established the current international dose standard, demonstrating that high‐dose radiotherapy (64.8 Gy) provided no advantage in OS, local control, or absolute survival metrics compared with the 50.4 Gy regimen [30]. This evidence supports the recommended definitive radiation dose range of 50.0–50.4 Gy in the international guidelines. However, the applicability of these findings in contemporary practice is limited by the trial's use of outdated two‐dimensional radiotherapy techniques. In contrast to these historical data, Chang et al.'s analysis of 2061 patients treated with modern CCRT and IMRT demonstrated significantly superior survival outcomes with higher radiation doses (≥ 60 Gy) compared with standard dosing (< 60 Gy) [31]. This discrepancy may be attributed to substantial differences in the pathological characteristics and tumor biology between Eastern and Western esophageal cancer populations. Consequently, a definitive radiation dose of 60 Gy remains the preferred approach in the Chinese clinical practice, reflecting these distinct disease characteristics and underscoring the need for population‐specific treatment protocols.

Several recent studies have evaluated the prognostic value of EDRIC in esophageal cancer, providing context for our findings. Cai et al. retrospectively analyzed 146 patients treated with CCRT and reported that an EDRIC ≥ 10.3 Gy was associated with a higher incidence of Grade 4 lymphopenia (55.2% vs. 4.5%) and poorer OS (HR = 1.142) and PFS (HR = 1.121) [32]. Zhao et al. evaluated 182 patients receiving neoadjuvant chemoradiotherapy for locally advanced esophageal squamous cell carcinoma and found that an EDRIC ≤ 6.86 Gy significantly improved disease‐free survival (HR = 0.37) and OS (HR = 0.22), with EDRIC closely correlated with lymphocyte nadir [33]. The optimal cutoff values vary across studies (10.3, 6.86, and 7.23 Gy in our cohort), which may be attributed to differences in total radiation dose (definitive doses of ≥ 60 Gy vs. neoadjuvant doses of 40–50.4 Gy), fractionation schedules, and planning target volume. Despite the variation in cutoff values, all three studies consistently demonstrate that a higher EDRIC is an independent predictor of poorer survival and is associated with severe lymphopenia. Collectively, these findings reinforce the potential of EDRIC as a quantifiable biomarker of radiation‐induced immune damage across different therapeutic modalities in esophageal cancer.

This study has several limitations. First, its retrospective, single‐center design introduces selection bias and unmeasured confounding. Second, the radiotherapy‐alone subgroup had a small sample size (*n* = 29, 17.5%), limiting the statistical power to assess the prognostic effect of EDRIC in the absence of chemotherapy. Third, EDRIC does not directly quantify the radiation dose received by the entire immune system. It is calculated from radiotherapy parameters and does not account for doses to immune‐related organs, such as lymph nodes and the spleen; therefore, it should be considered an estimate rather than a precise dosimetric biomarker. Fourth, lymphopenia during radiotherapy is multifactorial, influenced by radiation parameters, chemotherapy, nutritional status, and individual patient factors. Despite adjustment for treatment modality and the use of interaction analyses, residual confounding cannot be excluded. Finally, the median EDRIC in our cohort (7.23 Gy) was higher than previously reported values, which may be attributable to our prescribed dose range (50–70 Gy) and differences in the scanned body volumes across centers. Therefore, larger, prospective, multicenter studies are required to validate the prognostic value of EDRIC in esophageal cancer.

Nevertheless, the development of effective models to evaluate the radiation dose received by the immune system is crucial. Greater attention should be given to immune system damage associated with conventional radiotherapy modalities. During the implementation of personalized radiotherapy, a comprehensive assessment of the patient's physical condition, immune function, and disease status should be conducted to minimize radiation‐induced damage to the immune system, preserve immune function, and prolong patient survival.

## 5 Conclusion

EDRIC serves not only as a prognostic marker in patients with esophageal cancer undergoing radical radiotherapy but also as a potential biomarker of radiation‐induced lymphopenia and, more broadly, immune system damage.

## Funding

This work was supported by the Natural Science Basic Research Program of Shaanxi (2022JQ‐847).

## Ethics Statement

This study was performed in line with the principles of the Declaration of Helsinki. Approval was granted by the Ethics Committee of Second Hospital affiliated Xi'an Jiaotong University (approval number 2023551, date of approval 2024‐01‐30). Due to the retrospective nature of this study, the Ethics Committee of Second Hospital affiliated Xi'an Jiaotong University waived the requirement for written informed consent.

## Conflicts of Interest

The authors declare no conflicts of interest.

## Supporting information


**Table S1:** Interaction analysis between EDRIC and treatment modality for OS and PFS.

## Data Availability

The data that support the findings of this study are available on request from the corresponding author. The data are not publicly available due to privacy or ethical restrictions.
