# Supplementary material for: The Prognostic Value of EDRIC and Its Association With Radiation‐Induced Lymphopenia in Patients With Esophageal Cancer Undergoing Radical Radiotherapy: A Single‐Center Chinese Study
Source: Kaohsiung J Med Sci. 2026 Jun 11:e70251. Online ahead of print. doi: 10.1002/kjm2.70251 (PMC13399637; doi:10.1002/kjm2.70251)
Supplement: Supplementary file 1 — Table S1: Interaction analysis between EDRIC and treatment modality for OS and PFS. [file KJM2-9999-e70251-s001.docx]

Table S1. Interaction analysis between EDRIC and treatment modality for OS and PFS

| Endpoint | Interaction term | B (SE) | *P* value | HR (95%CI) |
| --- | --- | --- | --- | --- |
| OS | EDRIC × treatment | –0.019 (0.516) | 0.971 | 0.981(0.357–2.699) |
| PFS | EDRIC × treatment | –0.136 (0.480) | 0.777 | 0.873(0.341–2.238) |
